# Access to Medicines for Parkinson's Disease in Kenya: A Qualitative Exploration

**DOI:** 10.1002/mdc3.14192

**Published:** 2024-08-21

**Authors:** Natasha Fothergill‐Misbah, Juzar Hooker, Judith Kwasa, Richard Walker

**Affiliations:** ^1^ Population Health Sciences Institute Newcastle University Newcastle upon Tyne UK; ^2^ Aga Khan University Hospital Nairobi Kenya; ^3^ Department of Medicine University of Nairobi Nairobi Kenya; ^4^ Northumbria Healthcare NHS Foundation Trust Newcastle upon Tyne UK

**Keywords:** access, availability, affordability, medication, Africa

## Abstract

**Background:**

The accessibility of Parkinson's disease medicines is limited across sub‐Saharan Africa, which can have negative health, social and financial consequences for people with Parkinson's disease and their families. However, there is a stark gap in the literature regarding the impact of poor access to medicines on individuals.

**Objectives:**

The study objective was to understand the accessibility of Parkinson's disease medicines in Kenya from the perspective of people with Parkinson's disease, their caregivers and neurologists.

**Methods:**

In‐depth qualitative interviews were conducted with 55 people with Parkinson's disease, 23 caregivers and 8 neurologists to understand their experience regarding challenges with accessing Parkinson's disease medicines and the health, social and financial impact of poor availability and affordability.

**Results:**

Medicines for Parkinson's disease were deemed to be largely unavailable and unaffordable across Kenya. People with Parkinson's disease, caregivers and neurologists expressed the financial burden caused by long‐term treatment in the absence of health insurance coverage. Further, barriers accessing medicines negatively impacted symptom control, social relations, and quality of life.

**Conclusions:**

Access to Parkinson's disease medicines in Kenya is limited, with severe implications for symptom management and quality of life. People with Parkinson's disease should be able to access and afford the medicines they need to manage their condition.

The growing burden of Parkinson's disease (PD) has resulted in a pressing need for a global public health response.^1^ This is particularly the case for low‐ and middle‐income countries of the world, where the challenges of addressing the health and social requirements of people with PD are manifold.^2^, ^3^, ^4^, ^5^, ^6^ This paper focuses on the significant treatment gap that exists for PD, defined as the discrepancy between individuals living with PD and those receiving appropriate treatment, and the limited access to effective, affordable medicines experienced in Kenya.

Levodopa/carbidopa remains the most effective and widely available medicine for the symptomatic treatment of Parkinson's disease (PD) globally.^2^, ^7^ However, the poor accessibility of PD medicines, including levodopa/carbidopa, is a significant challenge for persons with PD (PwP) and their families across sub‐Saharan Africa. Challenges with accessibility result from several factors, including, but not limited to, poor awareness about PD,^8^, ^9^, ^10^, ^11^ the poor availability and affordability of medicines,^12^, ^13^, ^14^ poor health financing,^5^ lack of prioritization of neurological disorders in general,^6^ and the inefficiency of supply chains.^15^

The World Health Organization (WHO) 2nd Edition Neurology Atlas determined that just 3% of countries in the WHO African Region had levodopa/carbidopa always available at the primary care level.^5^ The unavailability and unaffordability of PD medicines has also been demonstrated through pharmacy surveys in Kenya,^13^ Nigeria^12^ and Ghana,^16^ and from a continent‐wide survey of neurologists in Africa.^17^ Due to the low coverage of health insurance schemes across sub‐Saharan Africa, PD medicines are paid for out‐of‐pocket. Therefore, affordability is a crucial component of accessibility, determined by the price of medicines and the financial capacity to pay for medicines for the duration of the condition, ie, life‐long.

Anecdotal evidence points towards the significant burden that a lack of access to medicines can cause for PwP and their families in sub‐Saharan Africa. However, data regarding the experience of these challenges are scarce. Therefore, the aim of this study is to explore the accessibility of PD medicines from the perspective of PwP, their caregivers and neurologists in Kenya, drawing on in‐depth interviews. Key research questions are:

1. What challenges do PwP and their families experience in accessing medicines for PD?
2. How do PwP and their families negotiate these challenges?
3. What are the outcomes of limited access to medicines for PwP and their families?

## Methods

Data presented come from a larger qualitative study on the lived experience of PD in Kenya carried out in 2018/19^4^, ^8^, ^18^, ^19^ which involved in‐depth semi‐structured interviews with patients, caregivers and healthcare professionals, and participant observations^20^ in neurology clinics and PD support groups. The aim of the wider study was to understand experiences of PD treatment, care, and support from diagnosis to the end of life.

Ethical approval for this study was obtained from Newcastle University Research Ethics Committee, Kenya Medical Research Institute (KEMRI) and hospital Institutional Review Boards (see “Ethical Compliance Statement”).

### Data Collection

This study focusses on interviews conducted with PwP (*n* = 55), family members/caregivers (*n* = 23) and neurologists (*n* = 8). Note: two of the interviewed neurologists are also co‐authors.

PwP and their caregivers were identified through three neurology clinics and a support group. PwP attending the clinics were invited to participate in the study with the permission and assistance of the neurologists and clinic staff. At the support group, PwP and caregivers were given information about the study and invited to approach the researcher if they were interested in taking part. Eight full‐time practicing neurologists who worked across the public and private sectors, exclusively in Kenya, were also invited to an interview.

All potential participants were given an information sheet to read (or requested that it be read to them). Informed, written consent was obtained from all PwP and caregivers. Neurologists gave verbal consent. All interviews were carried out by one interviewer in a place convenient for the participants and lasted 30–135 min. Most interviews were carried out in English. Nine interviews were in Kiswahili and with an interpreter to ensure accurate translations and limit loss of meaning.

### Data Analysis

Analysis of qualitative interview data followed Braun and Clarke's six phases of inductive thematic analysis,^21^ allowing themes to be identified from the data. Audio recorded interviews were transcribed and handwritten notes were typed up by the researcher. Coding was carried out line‐by‐line and conceptualized empirical and theoretical codes were collated into sub‐ and main themes. 10% of interview material was coded by a second experienced qualitative researcher who was not involved in the data collection to ensure reliability and to check for subjectivity. Continuous reflexivity throughout the process allowed for reflection on experiences and emerging themes. Verbatim quotes in the results are representative of wider responses. Pseudonyms are used for all participants.

The qualitative transcripts generated and analyzed in this study are not publicly available as participants did not consent to sharing of the raw data and doing so could compromise anonymity and confidentiality. Participants did, however, consent to anonymized verbatim quotes being used in publications. Data are available from the corresponding author on reasonable request.

## Results

The results presented are from interviews with 55 PwP (32 male and 23 female), 23 caregivers (7 male and 16 female) and 8 neurologists. Most PwP and caregivers lived in urban areas (*n* = 58) and fewer lived rurally (*n* = 20). All 8 neurologists were practicing in cities. PwP and families had different social and financial resources, ascertained through questions about employment, pension status and available basic and household amenities (eg, electricity) (Table 1). 70% of PwP had 7 or more of the 8 basic/household amenities, 3 PwP had 2 of the listed amenities, and 1 PwP had just 1 of the amenities (a radio).

**TABLE 1:** Characteristics of respondents

		% of PwP (yes)
Basic amenities	Electricity	92.5
	Sanitation	88.7
	Running water	88.7
	Flush toilet	71.7
Household amenities	Radio	98.1
	Sofa	96.2
	Television	88.7
	Car	49.1
Pension scheme		37.7
Currently employed		35.8

Median age of PwP was 66.5 years (range 33–81 years). Time since diagnosis ranged from 1 month to 18 years, although PD was often already quite advanced at diagnosis.^4^ Caregivers included daughter (*n* = 12), son (*n* = 6), wife (*n* = 4) and nephew (*n* = 1).

Fifty PwP (91%) had been prescribed levodopa/carbidopa (45 were using branded levodopa/carbidopa and 5 were using generic). Twenty‐one PwP (38%) were taking medicines in combination. Eight PwP (15%) were taking anticholinergics (trihexyphenidyl) which were widely available at low cost.

Martin, a 65‐year‐old PwP, had been diagnosed 3 years ago at the government neurology clinic. His wife had left him because of his condition. He had stopped working and relied on well‐wishers for food and medicines. Martin was prescribed five medicines (including levodopa/carbidopa 250/25 and trihexyphenidyl for his PD, an antidepressant, and medicines for diabetes and hypertension). Martin could not afford his PD medicines and often spent weeks without treatment, unable to get out of bed. Martin strategically sourced medicines to carry out necessary activities, such as to attend his neurology clinic appointment.

The costs of medicines, in addition to consultation fees and inpatient visits, were described as financially devastating by PwP and their families, who were forced to hold fundraisers, borrow money or sell assets to pay for PD medicines. For example:

*“The condition is continuing. So, you drain, you drain, you drain [resources], borrow, borrow, borrow [money]. You have not paid the money back and the drug is finished again. We have cows, we have sold them. You sell a plot of land. We have been drained. If at the month's end you don't have $400, he [PwP] won't survive.”* (*Spouse A, caregiver*)

Due to the high costs of medicines, PwP described taking their doses sporadically, stopping for periods of time, or altering their doses themselves because they could either not afford to take the medicines as prescribed, or could not source the medicines.

Gideon, a 33‐year‐old PwP, was diagnosed 3 years previously. He had two young children and had lost his job due to his condition. Gideon was prescribed levodopa/carbidopa 100/10 and amantadine. Due to the high costs of medicines and his reduced financial capacity, his neurologist removed amantadine from his prescription. Gideon eventually stopped taking the levodopa/carbidopa as well. Gideon prioritized his children's school fees and feeding his family over his medicines. His symptoms progressed uncontrolled, and he was often bedbound, could not work, worried about his and his children's futures, and described having suicidal thoughts. The challenge of altering doses based on what PwP could afford was also addressed by neurologists.

*“They decide maybe they don't increase the dose; they just take a little because that's what they can afford. The doctor increases the dose, the patient doesn't increase. The doctor adds another medication, the patient doesn't do it because it's going to cost them a lot of money. They use the same dose all along and they don't improve.”* (*Neurologist A*)

The neurologist cited here suggests that PwP only altered or rationed their medicines based on affordability, however, poor availability and accessibility were also common challenges cited by participants, particularly at government facilities. Families described spending days searching across cities to source PD medicines. For example:

*“There's a time last year, after getting the prescription, I went to one private hospital, finished, all of it. Then I went to another private hospital, there was none. As in, in a day, I did nothing, from morning to evening I just looked for Parkinson's medicines.”* (*Daughter A, caregiver*)

Several clinic files at the government hospital had “non‐compliant” written in them. However, this non‐compliance likely resulted from a combination of structural barriers, rather than the individual not wanting to take their medicines.

*“It's very difficult to get levodopa/carbidopa. In fact, one month ago we missed it completely and the patient went down as far as drooling and couldn't eat. The money you have is little, the drug cannot be found. We called Nairobi, they said there's none. We went around Mombasa, there was no chemist that sells it. If when you get $20 you can go to a shop and buy, still that would be better. But you get $100 or $150, you want the drug, you call Nairobi, none. You call Mombasa, none. Then what do I do?”* (*Spouse B, caregiver*)

Prescriptions were one of the few resources PwP were able to receive from neurologists,^4^ yet medicines were unavailable and unaffordable. Consequently, some PwP described using alternative healing methods, particularly when they were underdosed or not taking their medicines consistently or as prescribed, leading to ineffective management, the return of symptoms and a perceived ineffectiveness of pharmacological treatment. However, it was also because of the reluctance to admit that PD could not be cured by standard western pharmacological approaches to medicine, and the offering of a cure by alternative healers. Costs associated with alternative treatment also added to the financial burden of PD.

*“You know, when somebody is sick and you hear there is something which can help him recover, you listen attentively and the following morning you visit because you never know… We tried all we could to get him better”*. (*Spouse C, caregiver*)

Three caregivers discussed sourcing generic medicines from abroad (eg, India) at a fraction of the cost of purchasing them in Kenya. For example, paying $200 for 1 year's supply of generic medicines bought in India compared to $150 for 1 month's supply of originator medicines bought in Kenya.

Challenges with accessibility significantly impacted PwP and their families’ lives:

*“After I stopped [my medication], my symptoms got worse. I couldn't even get out of bed. I couldn't walk. I couldn't afford the medicines, and my family wasn't able to support me. I can't manage to pay school fees for my son and also go to hospital”*. (*PwP*)

Symptoms progressed untreated, resulting in disability and the continuous requirement for more care. PwP were unable to get out of bed, walk, get dressed or speak when they had not taken their medicines. For example, the family of one PwP were unable to source his medicines for five months—at an advanced disease stage and without any symptomatic treatment, he died from complications. His nephew explained, *“There was nothing I could do and nothing I could say. I didn't have any money. If there was medication he would still be here”*. Limited access to medicines and the inability to effectively manage PD symptoms also perpetuated stigmatizing perceptions, particularly those associated with supernatural beliefs.

## Discussion

This study explored the impact of poor accessibility of PD medicines in Kenya from the perspective of PwP, caregivers and neurologists, providing further evidence of limited availability and affordability, and novel data on the impact on individuals and their wider social networks, including symptomatic and financial suffering. Further, the narratives of PwP and caregivers provide a brief glimpse into the potentially desperate situation of those in Kenya, and elsewhere, who are never diagnosed and may receive no treatment at all throughout the duration of disease.

The study findings build on data regarding the unavailability and unaffordability of PD medicines across Africa.^12^, ^13^, ^16^, ^17^, ^22^, ^23^ Studies using WHO/Health Action International (HAI) methodology determined the costs of levodopa/carbidopa to be unaffordable across Africa (defined as paying more than one day's wage of the lowest paid government worker for a 30‐day supply). In Kenya, one month's supply cost $28–82,^13^ in Nigeria between $27–45^12^ and in Ghana between $35–71.^16^ A survey of neurologists across Africa also determined that PD‐specific therapies are largely unaffordable in most African countries.^17^ These data are supported by the narratives of PwP and caregivers in this study, who described the lived realities of the monthly and increasing costs of medicines as PD progressed. Out‐of‐pocket healthcare payments, such as for medicines, were shown to push more than 1 million people into poverty in Kenya in 2018.^24^

Participants also identified issues with the cost of originator medication in Kenya. Previous findings from Nigeria identified only branded/originator levodopa/carbidopa medication was available in pharmacies surveyed.^12^ It is well known that generic medicines are available at cheaper costs globally and it is therefore important that generic medicines are made available to PD populations to prevent high out‐of‐pocket costs.

The availability of medicines in pharmacies was a challenge cited by participants. This reiterates findings from WHO/HAI surveys which determined low availability of levodopa/carbidopa across public and private facilities (50% in Kenya (in 2014),^13^ 59% in Nigeria^12^ and 11% in Ghana^16^). Availability in these studies was particularly low in public facilities. In Kenya, availability in public pharmacies was 6.7%, compared to 73.7% in private pharmacies, where costs are also higher. Levodopa/carbidopa 100/25 (4:1 ratio) was one of the least available, and most expensive, formulations in all three surveys.^12^, ^13^, ^16^ Levodopa/carbidopa 10:1 ratio (ie, 250/25 or 100/10) can result in lower efficacy and more severe side effects, such as dyskinesias, potentially resulting in discontinuation.

15% of PwP were taking anticholinergics in this study. Anticholinergics are available and affordable in Africa and are commonly prescribed to PwP because of their accessibility.^12^, ^13^, ^25^ Though anticholinergics can help reduce tremors, they can cause confusion, hallucinations, and memory loss, in addition to constipation and dry mouth, particularly in older people. Anticholinergics also cannot act as a substitute for dopaminergic therapy.

Poor access to medicines contributed to unscheduled and/or improper dosing, poorly managed symptoms, and worsening motor and non‐motor symptoms, affecting quality life and increasing care requirements. It was difficult for PwP to effectively “self‐manage” their PD, something that is critical for the long‐term management of chronic conditions.^26^, ^27^ These challenges have been demonstrated in the management of other chronic conditions in Kenya, for example, diabetes.^28^ The role of family support in disease management is crucial in contexts where formal support and services are lacking.^29^ However, with catastrophic out‐of‐pocket spending on medication and absent health financing or social finance schemes,^30^ diseases such as PD can be devastating for families.

Further, PwP and families experienced stigmatizing perceptions, previously described in Kenya and across Africa.^8^, ^9^, ^10^, ^11^ If a disease cannot be cured by western medicine (such as taking a short course of antibiotics for treating an infection), there is more of an inclination to think that the PwP has been possessed by witchcraft or supernatural forces. Due to this misconception, some PwP may be isolated and/or excluded from their communities. PwP need to receive proper counseling about medicines, including side effects, risks of stopping and why alternatives may not be effective.

The challenges described in this paper occur despite levodopa/carbidopa 100/10 and 250/25 being included on Kenya's National Essential Medicines List 2023,^31^ which should mean that these essential medicines are available and affordable to those who need them.^32^ Only levodopa/carbidopa 250/25 is currently registered in Kenya by the national medicine regulatory authority. Other formulations (eg, levodopa/carbidopa 100/25), therefore, do not possess the license for sale in the country, creating issues with supply and preventing public procurement. Lack of registration of PD medicines has also been shown in neighboring Uganda and Tanzania.^33^ Table 2 outlines recommendations to improve access to essential medicines for PD in Kenya, and other countries in sub‐Saharan Africa facing similar challenges.

**TABLE 2:** Recommendations to improve access to medicines for PD in Kenya

Recommendation	Outcome
Update Kenya's National Essential Medicines List in line with updates to WHO's Model List of Essential Medicines and epidemiological data.	Levodopa/carbidopa in 4:1 ratio (100/25) listed as an essential medicine.
Register each essential medicine listed for PD with the national medicine regulatory authority, including diverse suppliers for each formulation and cheaper generics.	Facilitate legal import and sale of medicines for PD and improve availability in government facilities at lower costs.
Include essential medicines for PD on health insurance schemes.	Reduce out of pocket spending on PD medicines and improve affordability of medicines.

Promoting the accessibility of medicines for PD is a key action of the WHO Technical Brief “Parkinson Disease: a public health approach”,^2^ while the “Intersectoral Global Action Plan on epilepsy and other neurological disorders 2022–2031” (IGAP)^6^ and WHO report on ‘Improving access to medicines for neurological disorders'^34^ outline the need for sustainable access to affordable neurological medicines to reduce the treatment gap.^6^

This study was conducted in 2018/19, yet anecdotal evidence suggests the situation remains the same in Kenya, if not worse, due to increased costs of living and high inflation. Limitations of this study involve the inclusion of PwP (and their families) who had the social and/or financial resources to obtain a diagnosis, representing a more privileged group (see Table 1). Those who never receive a diagnosis and/or live in rural areas could have even worse outcomes without ever receiving treatment, or receiving treatment late in the course of the illness, as previously observed in Ghana.^35^ Having said that, this study contributes novel evidence regarding the situation of those who are affected by challenges with the accessibility of PD medicines, which has been missing from the literature from sub‐Saharan Africa.^11^, ^13^, ^17^

This study further highlights the necessity for PD medicines to be made available and affordable across Kenya, and particularly at government facilities, if PwP are to manage their condition well and live a good quality life. As work moves forward to generate awareness of PD and build a neurological workforce able to diagnose and manage PD across the continent, it is vital that this is accompanied by action to improve the accessibility of medicines.^4^

## Author Roles

(1) Research project: A. Conception, B. Organization, C. Execution; (2) Statistical Analysis: A. Design, B. Execution, C. Review and Critique; (3) Manuscript Preparation: A. Writing of the first draft, B. Review and Critique.

N.F‐M.: 1A, 1B, 1C, 3A, 3B

J.H.: 1B, 1C, 3B

J.K.: 1B, 1C, 3B

R.W.: 1A, 1B, 3B

## Disclosures

**Ethical Compliance Statement:** Ethical approval was obtained from Kenya Medical Research Institute (KEMRI) Scientific Ethics Review Unit (Ref: NON‐KEMRI 609), Newcastle University Research Ethics Committee (Ref: 1293/14933/2017), Kenyatta National Hospital‐University of Nairobi Ethics and Research Committee (Ref: P451/06/2018) and Aga Khan University Hospital Nairobi Institutional Ethics Review Committee (Ref: 2018/REC‐57). Approval to recruit from neurology clinics was given by clinic staff and neurologists. Written, informed consent was obtained from all participants by the researcher. We confirm that we have read the Journal's position on issues involved in ethical publication and affirm that this work is consistent with those guidelines.

**Funding Sources and Conflict of Interest:** This study was funded by the Economic and Social Research Council, United Kingdom (grant number ES/J500082/1). The authors declare that there are no conflicts of interest relevant to this work.

**Financial Disclosures for the Previous 12 Months:** The authors declare that there are no disclosures to report.
